# Retrograde Nailing for a Rare Post-Girdlestone Femur Fracture: A Case Report

**DOI:** 10.7759/cureus.77993

**Published:** 2025-01-26

**Authors:** Lamprini Agapitou, Stavros Lykos, Konstantinos Tsivelekas, Dimitrios Pallis, Stamatios A Papadakis

**Affiliations:** 1 Second Department of Orthopaedics, KAT General Hospital of Attica, Athens, GRC

**Keywords:** femur fracture, girdlestone, pji, resection arthroplasty, total hip arthroplasty

## Abstract

Femur shaft fractures are common in orthopedic practice, but their occurrence following Girdlestone resection arthroplasty (GRA) is extremely rare. We report the case of an unusual ipsilateral femoral shaft fracture after a ground-level fall in a patient who underwent hip resection arthroplasty nine years earlier due to prosthetic joint infection. The fracture was treated successfully with closed reduction and retrograde intramedullary nailing, enabling the patient to resume ambulation with pre-injury assistance levels. This case highlights the importance of understanding biomechanical implications in patients with GRA and demonstrates the efficacy of retrograde nailing in managing such rare fractures.

## Introduction

Periprosthetic joint infection (PJI) is a leading cause of failure in hip arthroplasty, often necessitating Girdlestone resection arthroplasty (GRA) [1]. The primary goals of GRA include eradicating infection and alleviating pain. In instances where a total hip prosthesis becomes infected with recurrent infections post-reimplantation, or when no further reconstructive options are viable, GRA may be considered a final alternative procedure [2]. While GRA is effective in reducing pain and eliminating infection, it is associated with complications such as leg shortening, abductor weakness, joint instability, and the onset of Trendelenburg gait [3,4]. Ambulation assistance is frequently needed, with reports indicating that up to 85% of patients require support [5]. Ipsilateral femoral fractures following GRA are rare, with limited references in the literature, likely due to the absence of the proximal fulcrum that typically prevents fractures of the femoral diaphysis [6,7]. This report presents a unique case of a total hip arthroplasty (THA), initially treated with GRA due to PJI, which subsequently resulted in a femoral diaphysis fracture after nine years of uneventful ambulation. We describe the mechanism of injury, the management strategy employed, and the outcome.

## Case presentation

**Figure 1:**
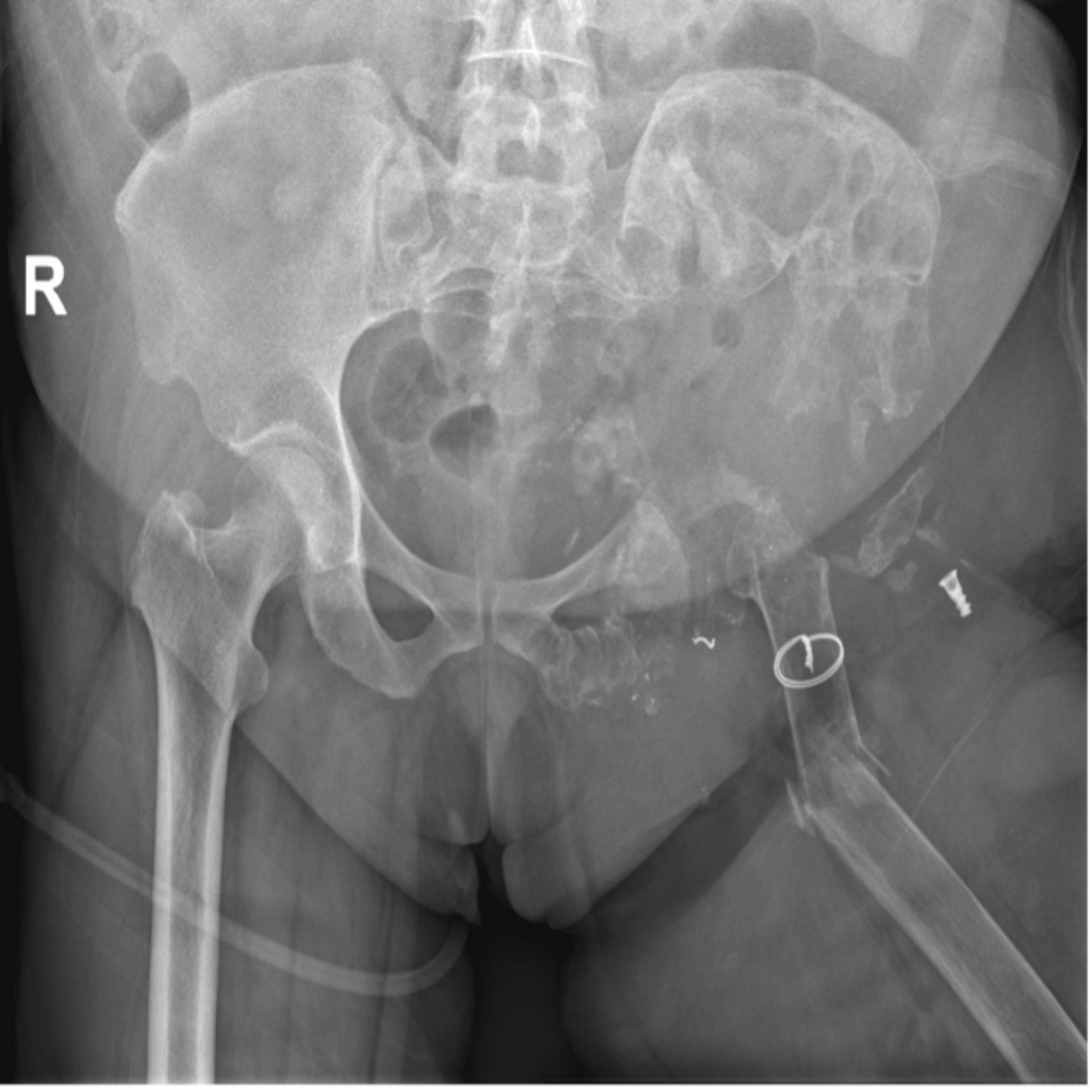
Pre-operative radiograph view of left femur shaft fracture

**Figure 2:**
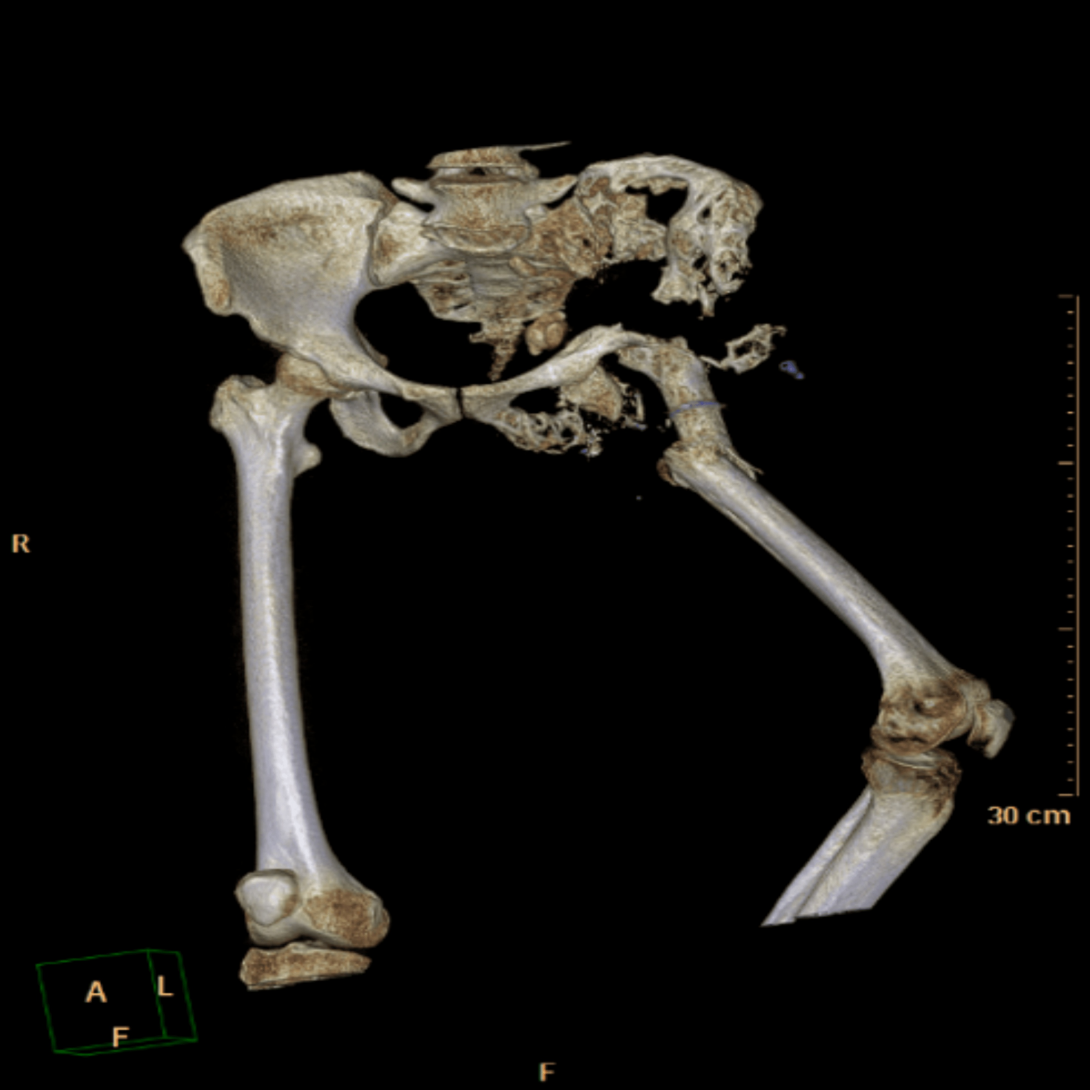
3D reconstructive image of the CT scan taken upon admission

A 64-year-old woman presented to the emergency department after a fall from standing height. She had a significant medical history, including developmental dysplasia of the hip, for which she underwent a THA 25 years ago. This initial procedure was followed by a revision THA 18 years later due to prosthesis loosening. Six years after the revision, she developed persistent left groin pain over several months and was subsequently diagnosed with chronic PJI caused by Staphylococcus epidermidis. A two-stage revision arthroplasty was performed, but the infection recurred a few months post-procedure. Due to the failure of multiple reimplantations, compounded by poor bone and soft tissue quality, she ultimately underwent a resection arthroplasty. She ambulated with assistance thereafter and resumed normal daily activities over the next nine years. The patient presented again after a slip and fall and was diagnosed with a left femur fracture distal to her resection arthroplasty (Figure 1 and Figure 2).

**Figure 3:**
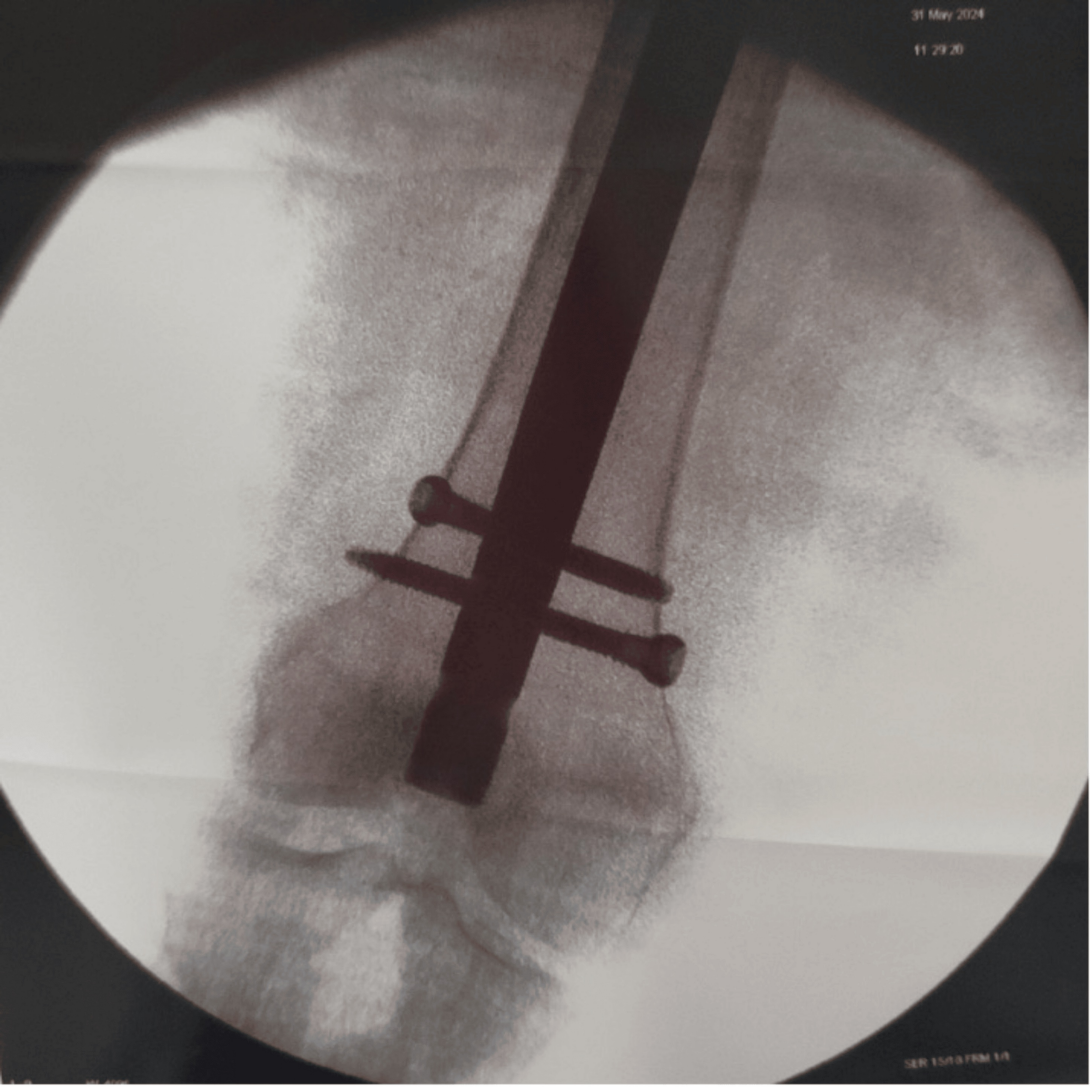
AP view of the intraoperative C-arm image showing the distal femur AP: Anteroposterior

**Figure 4:**
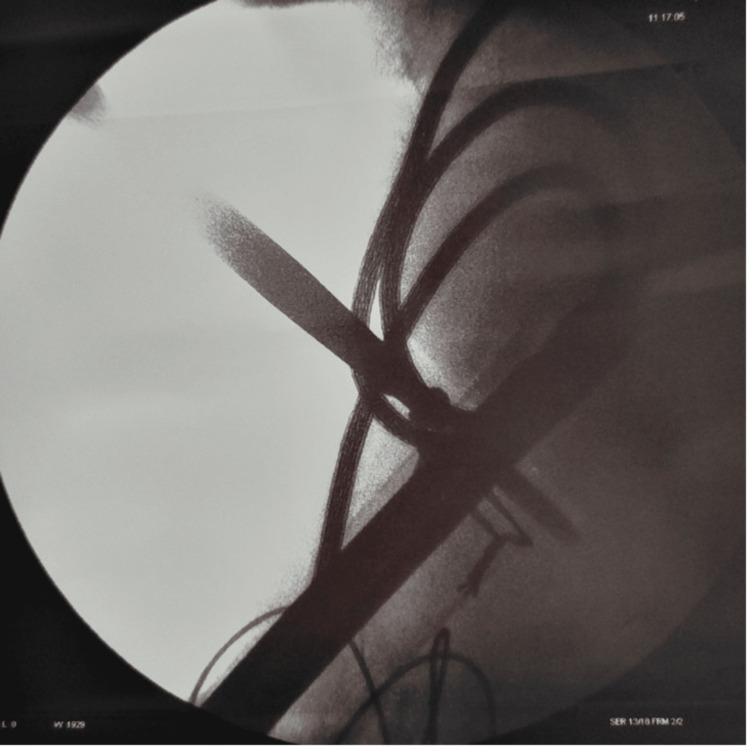
Lateral view of the intraoperative C-arm image showing the proximal femur

The fractured limb was immobilized and stabilized with a traction splint until surgery. The patient underwent closed reduction and intramedullary nailing for the left femoral fracture. An 11.5 mm × 28 cm locked retrograde femoral nail was inserted via an intercondylar notch start point. One proximal and two distal screws were used to interlock the nail. The position, placement, and reduction of the fracture were confirmed with a C-arm (Figure 3 and Figure 4).

**Figure 5:**
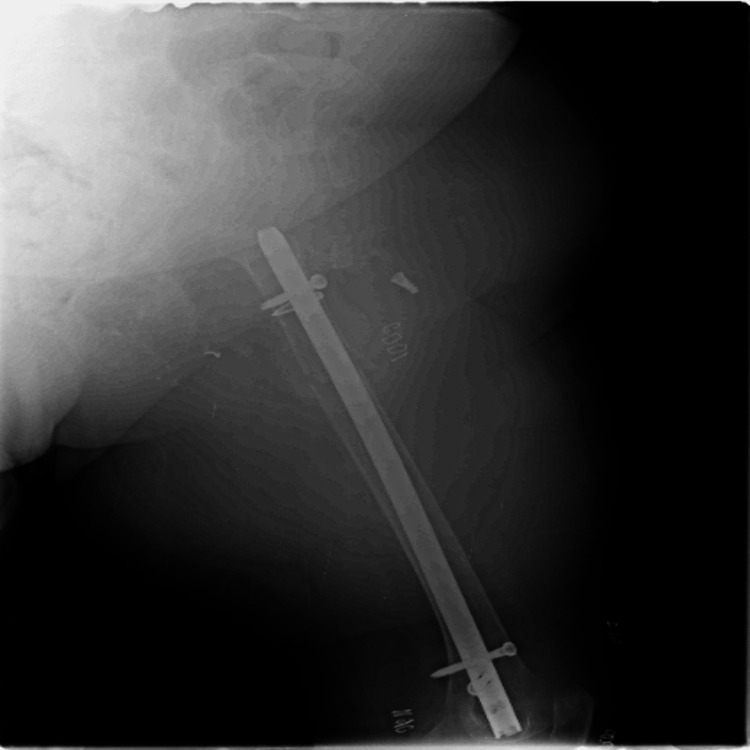
Post-operative lateral view of the femur shaft

The patient tolerated the procedure without complications and was discharged a few days later. At the first follow-up examination two weeks post-operatively, the stitches were removed, and a new X-ray confirmed satisfactory alignment (Figure 5).

## Discussion

The occurrence of a femoral shaft fracture after GRA is highly uncommon due to the altered biomechanics resulting from the loss of the proximal fulcrum. Factors related to the procedure and the patient’s condition likely contributed to this injury. During GRA, extensive curettage to eradicate infection can weaken the cortical bone, compromising its strength and increasing the risk of fractures [8,9]. In this case, significant bone loss during the excision of the proximal femur likely predisposed the patient to this fracture following a low-impact fall. Additionally, long-term changes such as osteoporosis due to disuse and underuse further weakened the femur, as has been observed in similar cases.

The management of femoral fractures following GRA presents unique challenges. Broadly, plate fixation is recognized for providing robust stabilization, particularly in complex fracture patterns, but it requires extensive soft tissue dissection, potentially disrupting the periosteal blood supply [10]. In contrast, retrograde intramedullary nailing offers distinct advantages, including shorter operative time, minimally invasive access, and better preservation of periosteal blood supply [6,7]. The use of interlocking screws further enhances construct stability, ensuring proper alignment and early mobilization.

The mechanism of injury and challenges in treatment reflect the altered biomechanics and weakened bone quality following GRA. The significant bone loss and reduced cortical integrity following curettage during the GRA procedure can predispose the femur to fractures, even with low-impact trauma [8,9]. Managing these fractures requires careful consideration of surgical techniques that minimize further bone damage and preserve the patient’s mobility.

This case demonstrates the importance of understanding the long-term biomechanical consequences of GRA, particularly in rare and complex fracture scenarios. Retrograde intramedullary nailing proved effective in this case, highlighting its viability as a treatment option for similar injuries. Further research is needed to better understand fracture patterns in patients with prior GRA and to develop standardized protocols for managing these rare injuries.

## Conclusions

This case report highlights the rare occurrence of ipsilateral femoral shaft fracture following GRA and underscores the challenges associated with managing such injuries. Retrograde intramedullary nailing proved to be an effective treatment option, enabling the patient to resume ambulation with pre-injury assistance levels. This outcome demonstrates the importance of careful biomechanical considerations in treating fractures in patients with prior GRA. Further studies are warranted to better understand the long-term biomechanical consequences of GRA and to establish standardized treatment protocols for such rare cases.
